# A folate receptor beta-specific human monoclonal antibody recognizes activated macrophage of rheumatoid patients and mediates antibody-dependent cell-mediated cytotoxicity

**DOI:** 10.1186/ar3312

**Published:** 2011-04-08

**Authors:** Yang Feng, Jiayin Shen, Emily D Streaker, Michael Lockwood, Zhongyu Zhu, Philip S Low, Dimiter S Dimitrov

**Affiliations:** 1 Protein Interactions Group, CCRNP, NCI-Frederick, NIH, 1050 Boyle Street, Frederick, MD 21702, USA; 2 Department of Chemistry, Purdue University, 560 Oval Drive, West Lafayette, IN 47906, USA; 3 BRP, SAIC-Frederick, Inc., NCI-Frederick, 1050 Boyle Street, Frederick, MD 21702, USA; 4 Indiana University Health Arnett Physicians, 2600 Greenbush Street, Lafayette, IN 47904, USA

## Abstract

**Introduction:**

Folate receptor beta (FRβ) is only detectable in placenta and limited to some hematopoietic cells of myeloid lineage in healthy people. Studies have indicated that FRβ is over-expressed in activated macrophages in autoimmune diseases and some cancer cells. In this study we aimed to develop an FRβ-specific human monoclonal antibody (mAb) that could be used as a therapeutic agent to treat rheumatoid arthritis and other autoimmune diseases, as well as FRβ positive cancers.

**Methods:**

Functional recombinant FRβ protein was produced in insect cells and used as antigen to isolate a mAb, m909, from a human naïve Fab phage display library. Binding of Fab and IgG1 m909 to FRβ was measured by ELISA, surface plasmon resonance, immune fluorescence staining, and flow cytometry. Antibody-dependent cell-mediated cytotoxicity (ADCC) was evaluated with FRβ positive CHO cells as target cells and isolated peripheral blood monocytes as effector cells in an *in vitro *assay.

**Results:**

Fab m909 bound with relatively high affinity (equilibrium dissociation constant 57 nM) to FRβ. The IgG1 m909 showed much higher (femtomolar) avidity as measured by ELISA, and it bound to FRβ positive cells in a dose-dependent manner, but not to parental FRβ negative cells. m909 did not compete with folate for the binding to FRβ on cells. m909 was not only able to select FRβ positive, activated macrophages from synovial fluid cells of arthritis patients as efficiently as folate, but also able to mediate ADCC in FRβ positive cells.

**Conclusions:**

Unlike folate-drug conjugates, m909 selectively binds to FRβ, does not recognize FRα, and has at least one effector function. m909 alone has potential to eliminate FRβ positive cells. Because m909 does not compete with folate for receptor binding, it can be used with folate-drug conjugates in a combination therapy. m909 can also be a valuable research reagent.

## Introduction

Folate (folic acid or vitamin B9) is essential for the biosynthesis of nucleotide bases and for many other methylation reactions. Not surprisingly, folic acid is required in increased amounts by rapidly dividing cells, such as cancer cells. In normal cells, folates are taken in through the reduced folate carrier (RFC) or proton-coupled folate transporter (PCFT), which are membrane-spanning proteins that facilitate bidirectional transportation of reduced folate across the plasma membrane and endosome membranes [1]. RFC is ubiquitously expressed in normal tissues and some tumors.

In addition to RFC and PCFT, a limited number of cells express folate receptors (FRs) that can mediate unidirectional transportation of folates into cells. Among the four isoforms of FRs identified (α, β, γ, and δ), α and β isoforms of FR are glycosylphosphatidylinositol (GPI)-anchored proteins with two N-glycosylation sites, and both have high affinity (K_D _of approximately 1 nM) for folate [2]. It is conceivable that FRs are useful when folate supply is low or when rapid cell growth requires elevated uptake of folate. Whereas FRα is expressed mainly in the apical surface of some polarized epithelial cells of normal tissues and on many cancer cells of epithelial origin [3], FRβ is limited mostly to placenta and some hematopoietic cells of the myelogenous lineage [4]. FRβ is also expressed on myelogenous leukemia (for example, acute myelogenous leukemia (AML) and chronic myelogenous leukemia) [2,5]. Although no FRβ-specific mAb has been studied in any clinical setting, a phase 2 trial (NCT00318370) has been completed for a humanized antibody against FRα (Farletuzumab) by Morphotek (Exton, PA, USA) to treat relapsed ovarian cancers after platinum chemotherapy [6]. Two more chimeric antibodies to FRα, MOv19 and MOv18, have been reported [7], and treatment of a xenograft mouse model with fusion protein of interleukin-2 and MOv19 single-chain variable fragment (scFv) has been shown to reduce the tumor volume [8].

A number of reports have shown that FRβ is present on activated macrophages that accumulate at sites of inflammation and in some tumors [9-11]. Resting macrophages, which are abundant in normal tissues and participate in homeostasis, have not been found to express FRβ. Resting macrophages can become activated by stimulation with cytokines or fragments of pathogenic microbes, resulting in the enhanced ability to kill and damage disease-causing microorganisms [12]. However, when activated inappropriately such as in autoimmune diseases, macrophages can cause severe tissue damage. Activated macrophages have been reported to be part of, but not limited to, important mechanisms in the following diseases: rheumatoid arthritis, lupus, atherosclerosis, psoriasis, diabetes, and transplantation rejection. Reports have shown that these activated macrophages in the intimal lining and sublining layer of synovial tissues from rheumatoid patients have receptors for folate, which are not present on resting macrophages [5,10]. Mouse peritoneal macrophages recruited by sublethal injection of live *Pseudomonas aeruginosa *have FRβ expression, whereas other cell populations, granulocytes, lymphocytes, or erythrocytes do not [5]. In rodent arthritis models, targeting activated macrophages with folate conjugates attenuates systemic and peri-joint inflammation and bone degradation [13,14]. Furthermore, the abundance of activated macrophages in rheumatoid arthritic joints, as measured by the uptake of a folate-linked imaging agent, could be related to the degree of articular inflammation [15]. In addition to infiltrating autoimmune and inflammatory diseases, macrophages infiltrate solid tumors, promoting tumor growth and metastasis by secreting proangiogenic factors and growth factors and by suppressing CD8^+ ^T cells. These tumor-associated macrophages have elevated levels of FRβ on their surface. The activated macrophages also have cell surface marker proteins (for example, CD86, CD80, and CD11b). It seems that, given the critical role of activated macrophages in autoimmune diseases and tumors, a therapeutic agent that targets these cells will have wide applications in the clinic. A substantial fraction of chronic myelogenous leukemia and AML cells also express FRβ [16,17].

In this study, we developed a fully human antibody, m909, specific to human FRβ (hFRβ), and demonstrated that this antibody is able to target FRβ-positive cells, including engineered cells as well as macrophages from rheumatoid patients, and induced antibody-dependent cell-mediated cytotoxicity (ADCC) of these cells. Therefore, m909 could be developed as a therapeutic candidate to treat the aforementioned autoimmune diseases and FRβ-positive tumors/leukemia.

## Materials and methods

### Expression of recombinant folate receptor beta

Human folate receptor beta (FRβ) fragment including amino acids 22 to 236 (the numbering is based on the sequence in NP001107007 in the National Center for Biotechnology Information database) was cloned from pcDNA3 to a baculovirus transfer vector pAcGP67 *via *SmaI and EcoRI sites. The primers used for the subcloning are 5'-cagtcccgggcaggacaggactgat-3' and 5'-gctggtgagatgcttcatcatcatcatcatcattgagaattcgact-3' (restriction sites underlined). The expression plasmid was co-transfected with BaculoGold viral DNA into SF9 insect cells in accordance with the instructions of the manufacturer (BD Bioscience, San Diego, California, USA). SF9 cells were infected with the high-titer viral stock for FRβ expression. Recombinant FRβ (rFRβ) protein was isolated from conditioned medium with a nickel-chelating column and was further purified with a Superdex75 10/300GL gel filtration column in PBS. The recombinant product had extra residues of alanine, aspartic acid, proline and glycine (ADPG) on the N-terminus and six histidines on the C-terminus. Purity of rFRβ was examined with 4% to 12% NuPAGE.

### Functional analysis of recombinant folate receptor beta

rFRβ was allowed to bind to Ni-NTA beads and was incubated with 0.1 μM folate-FITC (folate-fluorescein isothiocyanate) or folate-FITC solution and 100 μM unlabelled folate in PBS. After incubation for 1.5 hours at 4°C, the NTA bead slurry was centrifuged at 1,000 *g *for 3 minutes, and the NTA beads were washed with 20 mM imidazole buffer. The protein on NTA beads was released with 250 mM imidazole/PBS. The supernatant containing the eluted rFRβ was recovered and analyzed for fluorescence. Ni-NTA beads incubated with PBS were used as negative control.

### Antibody selection by phage display

Purified FRβ was used for panning of a human naïve Fab phage library in accordance with the protocol described in [18]. Three hundred colonies were picked from the last two rounds of panning and rescued with helper phage for screening. Two unique clones were selected for further affinity improvement by light-chain shuffling. Briefly, the heavy-chain sequence (NcoI and SpeI fragment) of the clone was gel-purified and ligated with the light-chain repertoire of the Fab library. The sub-library was further screened with rFRβ for three rounds. The clone with the best affinity, m909, was characterized here.

### Antibody expression and purification

The Fab fragment and IgG were prepared from HB2151 cells and 293Free Style cells, respectively, as described in [18]. Purified Fab has 6xHis and FLAG tags on its C-terminus.

### ELISA binding assay

rFRβ diluted in PBS was coated on a 96-well plate at 50 ng/well at 4°C overnight. Wells were blocked with 100 μL of 4% nonfat dry milk/PBS (MPBS) for 1 hour at 37°C. Antibodies were diluted at indicated concentrations, and each concentration was tested with duplicate wells at 50 μL/well. After 2-hour incubation at 37°C, the wells were washed four times with PBST (0.05% Tween 20 in PBS). Bound Fab was detected with anti-FLAG-HRP mAb (1:1,000) (Sigma-Aldrich, St. Louis, MO, USA) for 1 hour at 37°C. Wells were washed again with PBST, the substrate ABTS was added (50 μL/well), and the absorbance was read at 405 nm. For ELISA with IgG, a goat anti-human Fc IgG (Jackson ImmunoResearch Laboratories, Inc., West Grove, PA, USA) conjugated with HRP was used at 1:1,000.

### Surface plasmon resonance analysis

Binding of m909 Fab to human rFRβ was assayed by using a Biacore X100 instrument (GE Healthcare, Piscataway, New Jersey, USA). Purified rFRβ was diluted in 10 mM sodium acetate buffer (pH 5.0) and immobilized on a CM5 sensor chip with an amine coupling kit. The reference flow cell was treated with the amine coupling reagent without exposure to rFRβ. The running buffer was HBS-EP (10 mM HEPES, pH 7.4, 150 mM NaCl, 3 mM EDTA, 0.05% surfactant P20). m909 Fab, diluted with the running buffer, was allowed to flow through the cells. The chip was regenerated with 10 mM glycine (pH 2.5) and 1 M NaCl. The sensorgram was analyzed with BIAevaluation software (GE Healthcare), and data were fitted to a 1:1 binding model.

### Flow cytometry

CHOK1 cells (FRβ-negative) and CHO-hFRβ (expressing high levels of hFRβ on their surface) and preB L1.2 (having low levels of hFRβ surface expression) were analyzed in accordance with the procedure described in [19]. Flow cytometry was conducted with monocytes and macrophages isolated from patients: Synovial cells from patients with rheumatoid arthritis or monocytes from healthy donors were isolated with Ficoll gradient separation and were stained with the appropriate marker antibodies (anti-CD14, -CD16, or -CD11b) for 30 minutes on ice. Samples were washed three times with PBS, and this was followed by incubation with folate-Oregon Green (100 nM) for 60 minutes at 37°C or with 50 nM m909-FITC for 60 minutes on ice. In competition studies with folate-Oregon Green, cells were co-incubated with 10 μM unlabelled folate to competitively block all FR. Isotype control IgG was used as negative control for m909. Flow cytometry was performed on FACSCalibur, and CellQuest was used for data acquisition and analyses. The fluorescence gate for FR expression (x-axis) was set so that less than 1% of macrophages were counted as FR-positive in the presence of folate-Oregon Green plus 100-fold excess unlabelled folate. Similarly, the fluorescence gate for activation markers was set so that less than 1% of the macrophages appeared to be positive when examined with a nonspecific antibody isotype control. Experiments from each group were repeated at least three times, and representative data from each group are shown.

### Collection of synovial fluids from patients with rheumatoid arthritis

Rheumatoid arthritic synovial fluid samples were obtained from four patients whose rheumatoid arthritis was diagnosed at Indiana University Health Arnett (Lafayette, IN, USA). All procedures were approved by the institutional review boards of Purdue University and Lafayette Home Hospital and St. Elizabeth Medical Center. Patients were recruited to the study after informed consent.

### Confocal microscopy

hFRβ stably transfected CHO-hFRβ cells, CHO-K1 cells, and KB nasopharyngeal epidermoid cells were seeded in chambered coverglass wells and allowed to adhere for 24 to 36 hours in a 37°C incubator. Unattached cells were rinsed off with warm PBS, and attached cells were incubated with 50 nM FITC-m909 IgG for 1 hour at 37°C and then washed three times with cold PBS. KB cells were also incubated with an FRα-specific mouse mAb conjugated with FITC to show FRα expression on these cells. The binding of antibodies to cells was visualized with an IX81 inverted microscope (Olympus America Inc., Center Valley, PA, USA) equipped with an FV1000 confocal unit and a 60 ×/1.2 NA (numerical aperture) oil objective. A 488-nm argon laser was used to excite the FITC. The green color imaging was captured with the spectral detector, and the emission spectrum of fluorescein was monitored between 500 and 530 nm. Images were processed using FLUOVIEW software (Olympus America Inc.).

### Cell lysis by antibody-dependent cell-mediated cytotoxicity

Peripheral blood mononuclear cells (PBMCs) were isolated from healthy donors by means of Ficoll-Paque Plus (GE Healthcare). Collection of blood from donors was approved by the NCI-Frederick Research Donor Program. The viability of isolated cells was greater than 95%. PBMCs were seeded in a 96-well plate in RPMI, 10% fetal bovine serum at 500,000 cells per well. Cells were incubated at 37°C and allowed to attach to the wells for 3 hours. Unattached cells were rinsed off by two washes of warm PBS; cells attached to the wells were used as the effector cells. Target cells, CHOK1, CHO-hFRβ, or preB L1.2 cells, were trypsinized and resuspended into single-cell suspensions. The target cells were incubated with 8, 40, or 200 nM IgG m909 or control IgG at room temperature for 30 minutes and then added to effector cells at 10,000 cells per well. The ratio of effector to target cells was 50:1. The plate was centrifuged at 300 *g *for 5 minutes and incubated at 37°C for 24 hours. Supernatant (100 μL) was transferred to an all-white plate, and 100 μL of CytoTox-ONE reagent (Promega Corporation, Madison, WI, USA) was added to each well. The lactate dehydrogenase released from lysed cells converted the CytoTox substrate to fluorescent resazurin, whose signal was detected in fluorometer (excitation wavelength of 560 nm and emission wavelength of 590 nm). The percentage of specific lysis was calculated as follows: (experimental treatment-effector cell control)/(high control-target cell control) × 100%. Measurement of target cells alone treated with 1% Triton X-100 was used as the high control. Each treatment was carried out in six duplicate wells and each assay plate included control wells.

## Results

### Expression and functional analysis of recombinant folate receptor beta

**Figure 1:**
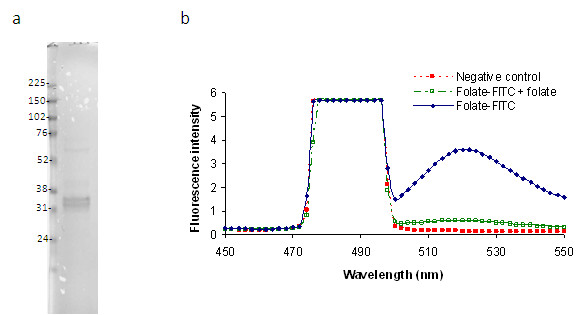
**Recombinant rFRβ is functional in binding folate**. **(a) **Purified rFRβ was resolved on 4% to 12% NuPAGE gel. The rFRβ appears as a tight doublet. Molecular weight markers (in kilodaltons) are on the left side of the gel image. **(b) **rFRβ is able to bind to folate. Open squares indicate NTA control with FITC-folate, filled squares indicate rFRβ with FICT-folate and 100-fold excess unlabelled folate, and filled diamonds indicate rFRβ with FITC-folate. FITC emission is detected at 518 nm. FITC, fluorescein isothiocyanate; rFRβ, recombinant folate receptor beta.

The hFRβ is a 255-amino acid membrane protein with a short signal peptide (amino acids 1 to 22) and a C-terminal transmembrane tail (amino acids 237 to 255), which forms the GPI anchorage. Thus, the part of FRβ from amino acids 23 to 236 represents its functional extracellular domain in the mature protein and would be ideal for using as bait in phage library screening. This fragment was designed for recombinant expression in insect cells using a baculoviral system. The recombinant FRβ (rFRβ) was purified from culture supernatant with a nickel-chelating column followed by Superdex75 column chromatography. The rFRβ ran as a tight doublet on reduced NuPAGE, most likely because of heterogeneous post-translational modifications such as glycosylation (Figure 1a). The doublet profile of folate-binding protein has been reported for chicken riboflavin-binding protein [20,21], which is closely related to FRβ. Importantly, rFRβ was confirmed to bind to its ligand, folate (Figure 1b). The purified rFRβ was then used in the following panning experiments.

### Selection of m909 from human naïve Fab phage display library with recombinant folate receptor beta

**Figure 2:**
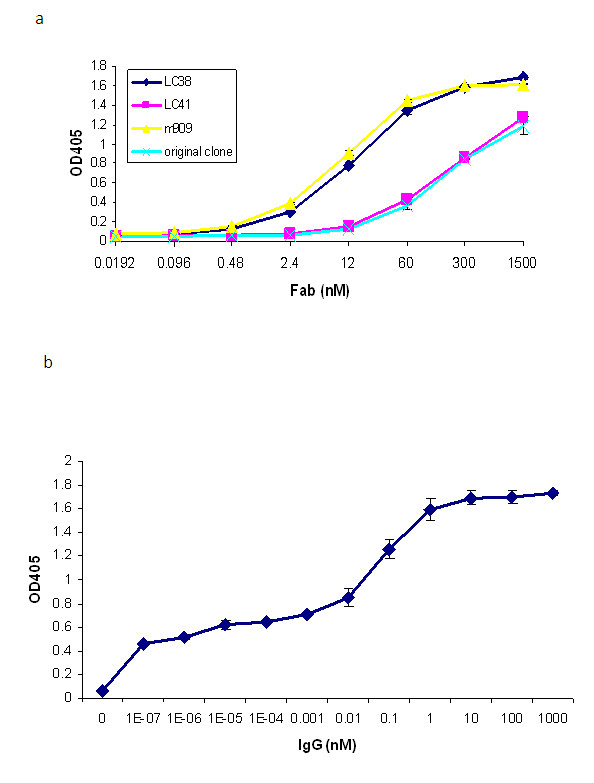
***In vitro *ELISA binding of anti-rFRβ Fabs and affinity-matured clone m909**. **(a) **The parental Fab clone and light-chain shuffled clones were tested on ELISA wells coated with rFRβ. All Fabs were purified from periplasm of *Escherichia coli *and tested from 1,500 to 0.0192 nM. m909 was the clone featured in the study. LC38 and LC41 are two other affinity-matured clones. **(b) **m909 IgG was tested for binding to rFRβ on ELISA. The highest IgG1 concentration tested was 1,000 nM and was serially diluted at 1:10. m909 IgG showed binding even at 1 fM. ELISA, enzyme-linked immunosorbent assay; Fab, antigen-binding fragment; OD, optical density; rFRβ, recombinant folate receptor beta.

**Figure 3:**
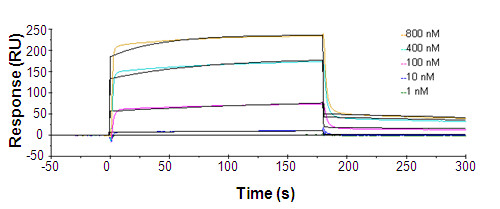
**Biacore analysis of m909 Fab**. rFRβ was immobilized on a CM5 sensor chip at 210 resonance units (RU). m909 Fab at 1, 10, 100, 400, and 800 nM was tested. A 1:1 binding model gave the equilibrium dissociation constant (K_D_) of 56.72 nM. The colored lines are the binding curves, and the black lines are the fitted curves. Fab, antigen-binding fragment; rFRβ, recombinant folate receptor beta.

An Fab clone was first selected from the human naïve Fab phage display library and had an estimated 100 nM half maximal effective concentration (EC_50_) to rFRβ on ELISA. This clone was affinity-matured using a light-chain shuffling method and rescreened with rFRβ under a more stringent set of conditions. A Fab clone with better affinity, m909, was selected from the maturation process (Figure 2a), and this is the antibody characterized in the present study. m909 was converted into both single-chain (scFv) and IgG1 formats for different applications. On ELISA, Fab m909 was shown to exhibit an EC_50 _of approximately 10 to 50 nM, whereas its IgG1 showed significant binding to rFRβ on ELISA even at femtomolar concentrations (Figure 2b), implying the importance of the avidity effect for its binding. It should be noted that m909 in either format did not bind to the α isoform of the human FR (data not shown). The binding kinetics of m909 Fab was further tested with the surface plasmon resonance Biacore instrument. Fab m909 has an equilibrium dissociation constant (K_D_) equal to 57 nM (k_a _= 2.793 × 10^4 ^1/Ms and k_d _= 0.001584 1/s) (Figure 3), which is in agreement with the estimation from the ELISA binding assay.

### m909 binds to native human folate receptor beta on cell surface

**Figure 4:**
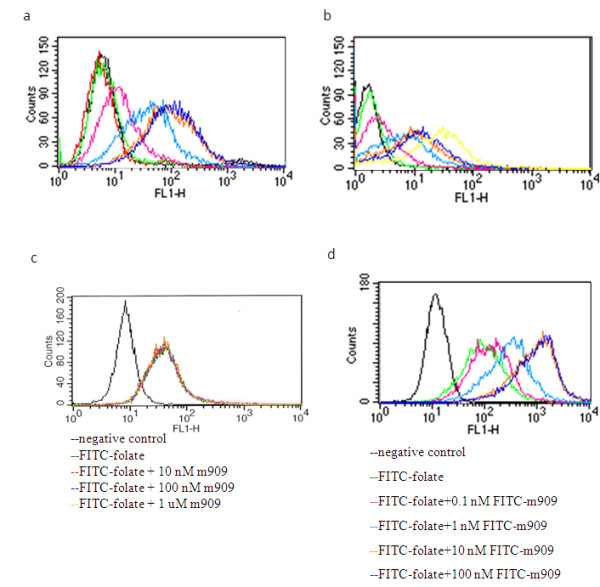
**Flow cytometry of m909 IgG on FRβ-positive cells**. hFRβ-positive cells were incubated with 0.001, 0.01, 0.1, 1, 10, and 100 nM m909 IgG. An isotype control IgG1 was included in the test at 100 nM. Cells were analyzed in FACSCalibur. **(a) **CHO-hFRβ cells. **(b) **PreB L1.2 cells. Black line indicates isotype control IgG, and colored lines indicate 0.001 to 100 nM m909. **(c) **CHO-hFRβ cells were incubated with 10 nM folate-FITC (green line) and 10, 100, and 1,000 nM unlabelled m909 (other colored lines). **(d) **CHO-hFRβ cells were incubated with 10 nM folate-FITC and varying concentrations of m909-FITC. Binding of m909 and folate did not interfere with each other. Black line indicates negative control, green line indicates FITC-folate alone, and other colored lines indicate FITC-folate with 0.1, 1, 10, or 100 nM m909-FITC. FITC, fluorescein isothiocyanate; FRβ, folate receptor beta; hFRβ, human folate receptor beta.

To further characterize the binding ability of m909 IgG1, we tested to see whether it might recognize native FRβ on the surface of the cell. This was investigated through flow cytometry and immunofluorescent staining of cells. CHO-hFRβ cells are engineered from parental CHO-K1 cells (FRβ-negative) and stably express relatively high levels of hFRβ on their surface. In flow cytometry, m909 IgG1 bound to CHO-hFRβ cells (Figure 4a) but not to CHO-K1 cells, indicating that the antibody recognizes FRβ specifically and does not recognize other membrane proteins on these cells. Another cell line, preB L1.2, has been stably transfected with FRβ but showed lower levels of FRβ expression (Figure 4b). When m909 and its isotype IgG were evaluated for binding by flow cytometry to these cells, dose-dependent binding was observed, albeit at a much lower intensity than that of binding to CHO-hFRβ cells. m909 did not bind to CHO-K1 cells at the highest concentration tested (data not shown).

It was also of interest to explore whether m909 might bind to the same site on FRβ as does folate. To answer this question, we incubated folate-FITC with CHO-hFRβ cells in the presence of varying concentrations of m909 IgG and found that the addition of unlabelled IgG1 m909 did not change the folate-FITC signal intensity (Figure 4c). Next, folate-FITC was co-incubated with CHO-hFRβ cells in the presence of increasing concentrations of FITC-labelled m909 IgG. It was found that the addition of m909-FITC increased the signal intensity over that of folate-FITC alone (Figure 4d), indicating that the bindings of folate and m909 are not mutually exclusive and that they have at least an additive effect, if not a synergistic one.

**Figure 5:**
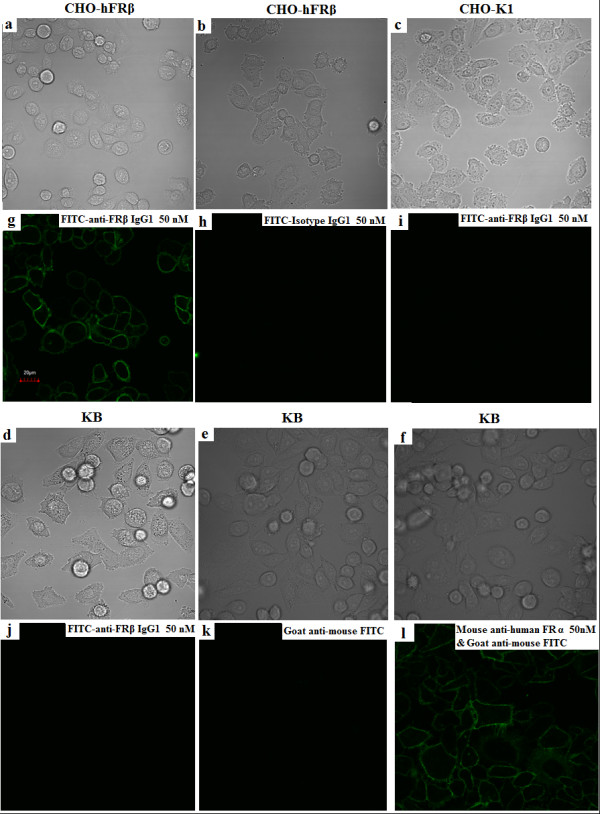
**Confocal laser microscopy images show the specific binding of FITC-m909 to CHO-FRβ cells**. Human FRβ stably transfected CHO-FRβ cells **(a,b,g,h)**, CHO-K1 cells **(c,i)**, and KB nasopharyngeal epidermoid cells **(d-f,j-l) **were incubated with 50 nM m909 IgG-FITC for 1 hour at 37°C and were washed three times with cold phosphate-buffered saline. Images (a-f) were captured with transmitted light. Images (g-l) were captured using a charge-coupled device camera with identical settings below the saturation limits. Isotype IgG1-FITC did not give any binding signal in CHO-FRβ cells (b,f). Mouse anti-human FRα mAb together with goat anti-mouse IgG-FITC secondary antibody showed the expression of human FRα on KB cells (f,l). FITC, fluorescein isothiocyanate; FR, folate receptor.

FRs are GPI-linked membrane proteins that are readily accessible to drugs, and this renders them potential targets for treatment of arthritis and cancers. To confirm that m909 can indeed bind to FRβ on intact cells, we investigated whether m909 binding could be visualized on cell surfaces. FITC-labelled m909 was incubated with CHO-hFRβ or CHO-K1 cells cultured in coverglass wells, and cells were examined by confocal microscopy to investigate the subcellular localization of the antibody binding. As shown in Figure 5, IgG1 m909 staining was found predominantly on the plasma membrane of CHO-hFRβ cells, and little staining was detectable inside cells. These results agree with the flow cytometry results and further indicate that receptor downregulation was minor under the experimental condition (37°C for 1 hour). The isotype control IgG1-FITC did not have any detectable staining in CHO-hFRβ cells, nor did m909 stain the parental CHO-K1 cells. KB nasopharyngeal epidermoid cells have been reported to display only the α isoform of FR on their surface [22]. Staining with a mouse mAb specific to FRα showed that KB cells have a significant amount of FRα on their surface (the last panel in Figure 5). IgG1 m909 failed to stain KB cells, indicating that it is specific for hFRβ, in agreement with the ELISA results.

### m909 binds to human folate receptor beta selectively on inflammatory monocytes and activated macrophages from synovial fluid of arthritis patients

Several reports have shown that activated macrophages and monocytes in autoimmune diseases have elevated levels of FRβ [5,11,23]. In addition, some solid tumors are infiltrated with macrophages, among which a high percentage are FRβ-positive [9,24]. These macrophages are capable of secreting cytokines, growth factors, and proangiogenic factors. Eliminating activated macrophages from autoimmune disease tissues and tumors could be beneficial to these patients. Therefore, we investigated whether m909 recognizes these diseased cells by isolating such cells from two sources.

**Figure 6:**
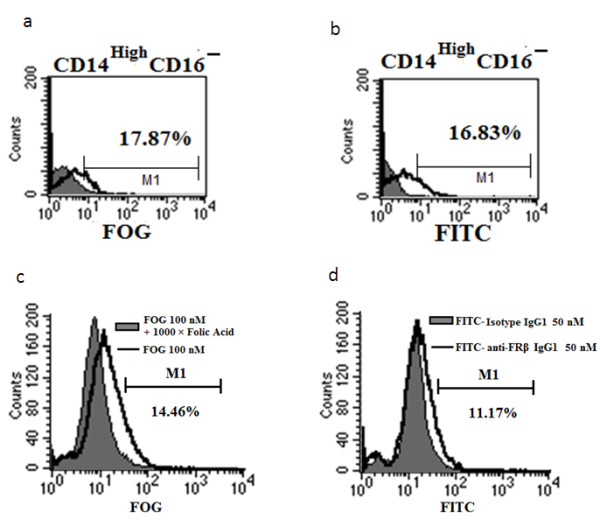
**m909 binds to FRβ**^**+ **^**CD14**^**high **^**CD16^- ^subset (inflammatory monocytes) of human PBMCs and activated macrophages from synovial fluid of rheumatoid arthritis patients**. Human PBMCs were stained with PE-anti-CD14 and Tricolor-anti-CD16 antibodies and **(a) **folate-Oregon Green (FOG) or **(b) **m909-FITC. (a) The cells were stained with 100 nM folate-FOG in the absence (solid black histogram) or presence of an excess (10 μM) of free folic acid to competitively occupy FR (filled gray histogram). (b) The cells were stained with 5 nM m909 IgG-FITC (solid black histogram) or 5 nM control IgG1-FITC (filled gray histogram). Among the CD14^high ^CD16^- ^cells, m909 selected 16.83% of cells and folate selected 17.87%. **(c,d) **Synovial fluid cells from four patients with rheumatoid arthritis were first labelled with anti-CD11b to stain human macrophages and then incubated with (c) 100 nM folate-FOG in the absence (unfilled histogram) or presence of an excess (100 μM) of free folic acid to competitively occupy FR (filled gray histogram) or (d) 50 nM m909-FITC (unfilled histogram) or 50 nM control IgG1-FITC (filled gray histogram). A representative flow plot with the percentage of FRβ-positive cells within each gate is shown. FITC, fluorescein isothiocyanate; FR, folate receptor; PBMC, peripheral blood mononuclear cell.

First, it has been observed that approximately 10% of the PBMCs of apparently healthy people express measurable numbers of hFRβ, perhaps in response to a low constitutive inflammatory process. To explore whether m909 might bind these monocytes, we separated CD14^high ^PBMCs into CD16^+ ^and CD16^- ^groups. The latter group of cells represents the myelomonocytic lineage cells with high levels of receptor for lipopolysaccharide (or endotoxin) but lacks FcγRIII (a hallmark of natural killer (NK) cells). We found that, among the PBMCs from this particular donor examined, approximately 17% of the CD14^high^CD16^- ^cells have FR on their surface, shown by incubation with 100 nM labelled folate (Figure 6a). Importantly, a similar percentage of these cells was found to be positive for hFRβ, shown similarly by incubation with 5 nM m909 IgG (Figure 6b). These results demonstrate that m909 selects FR-positive cells from monocytes as efficiently as folate at tested concentrations. Furthermore, this assay provides confirmation that it is hFRβ, not the hFRα, that is upregulated in the activated macrophages.

Next, we isolated synovial macrophages from four patients with rheumatoid arthritis. Previous studies have shown that synovial macrophages collected from patients with arthritis have elevated FRβ, and this hFRβ is able to internalize folate-conjugated drugs [5,10]. Activated macrophages were first selected with the CD11b marker. The subpopulation of cells was further analyzed for hFRβ expression either through folate-Oregon Green or m909-FITC. It was found that fluorescent folate can label approximately 14.5% of macrophages (Figure 6c), whereas m909 selected approximately 11.17% of activated macrophages (Figure 6d). Competition with 1,000-fold excess of non-labelled folate and an isotype control IgG1 were used in these tests to subtract background. These results indicated that m909 and folate are similarly effective in the selection of activated macrophages. Together, these data indicate that m909 specifically recognizes FRβ-positive inflammatory monocytes and activated macrophages from patients.

### m909 induces antibody-dependent cell-mediated cytotoxicity with human folate receptor beta-positive cells

**Figure 7:**
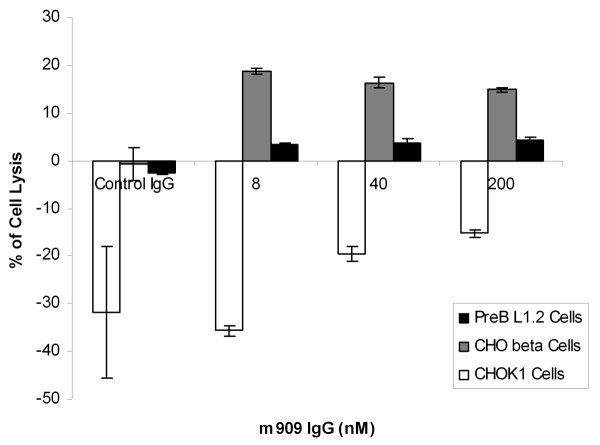
**m909 induces ADCC in FRβ-positive cells but not in FRβ-negative cells**. Freshly isolated PBMCs were incubated with target cells (CHO-hFRβ, PreB L1.2, or CHO-K1) at a ratio of 50:1 in the presence of m909 IgG1 at three different concentrations (8, 40, or 200 nM) or an isotype control IgG1 at 200 nM (first set of columns). ADCC was detected with CytoTox-ONE reagent, allowing measurement of the lactate dehydrogenase released by lysed target cells. The percentage of specific lysis was calculated as described in Materials and methods. Dark bar indicates preB L1.2 cells, gray bar indicates CHO-hFRβ cells, and clear bar indicates CHO-K1 cells. ADCC, antibody-dependent cell-mediated cytotoxicity; FRβ, folate receptor beta; PBMC, peripheral blood mononuclear cell.

During an initial attempt to examine whether m909 might affect the growth behavior of hFRβ-positive cells, we did not detect any impact of the antibody. However, because cells decorated with human IgG are often recognized and destroyed by NK cells, we decided to explore whether m909 might mediate ADCC. To test this possibility, PBMCs were isolated from healthy donors and incubated with CHO-hFRβ, CHO-K1, or preB L1.2 cells at a ratio of effector cells to target cells of 50:1. The IgG1 m909 or an isotype control IgG was incubated with cells at varying concentrations. IgG1 m909 was found to induce specific target cell lysis in an hFRβ level-dependent fashion; m909 induced significantly more lysis in CHO-hFRβ cells than preB L1.2 cells (Figure 7), which have positive but lower levels of FRβ than CHO-hFRβ cells. In the parental CHO-K1 cells, m909 did not induce any detectable cell lysis. The control IgG did not have cytotoxicity at 200 nM. It seems that cell lysis reached maximum at 8 nM IgG1 m909; this probably reflects the saturation of m909 binding on CHO-hFRβ cells at this level. The notion is supported by data in Figure 4a that 10 nM (the orange line) and 100 nM (the dark blue line) m909 IgG had almost the same binding. Together, these results indicate that IgG1 m909 bound to the cell surface was able to attract NK cells and thereby mediate specific cell killing by NK cells.

## Discussion

FRs have been the focus of studies for decades [25]. Only in the past 10 years have their roles in cancer treatment been actively researched. Among the studies reported to date, a majority of them focus on targeting FR with its ligand folate. Folate-drug conjugates have achieved considerable successes in diagnosis and treatment of many diseases, especially rheumatoid arthritis. These folate conjugates are designed to kill diseased cells through one of two mechanisms: direct surface targeting/binding or folate-activated receptor endocytosis.

Antibody-based therapeutics have advanced significantly in the past decade because of the recombinant antibody technologies. Several chimeric antibodies targeting the receptor FRα have been reported, and one such antibody, Farletuzumab, was studied in a phase 2 clinical trial [26]. At present, no human antibody specific to FRβ has been reported. A rat mAb to mouse FRβ was reported to reduce tumor-associated macrophages when its single chain was fused with immunotoxin and used to treat rat glioma in a nude mouse model [9]. The unique expression profile of FRβ in activated macrophages and AML supports the notion of targeting FRβ for treatment of autoimmune diseases and myelogenous leukemias. In AML cells, the level of FRβ can be upregulated by treatment with all-*trans *retinoic acid [27], thereby increasing the specificity of FRβ-targeted therapy.

In this study, we have produced the first functional recombinant hFRβ reported in the literature. m909 was selected from a human Fab library with rFRβ and was found to specifically recognize hFRβ but not the α isoform of FR, giving it an advantage when the binding of FRα is to be avoided. m909 is also capable of selectively binding to inflammatory monocytes and activated macrophages from the synovial fluid of patients with rheumatoid arthritis. Whereas the activated macrophages have elevated levels of FRβ, the normal residential macrophages do not express FRβ. Therefore, m909 is a good candidate for diagnosis and treatment of both autoimmune diseases that involve activated macrophages and tumors that are infiltrated with activated macrophages.

Many imaging and therapeutic agents using folate have been reported, as folate has advantages as a small molecule, being easy to produce and to conjugate to drugs, as well as having quick clearance from the circulation when used as an imaging agent [28]. However, in some cases, a receptor-targeted method is preferred. For example, some cells have FRs, but owing to changes in post-translational modifications, these receptors do not bind to folate [4]. Folate and its conjugates do not distinguish between the two isoforms of FR. When only FRβ, not the α isoform of FR, is required for targeting, m909 allows the specificity. Antibodies by nature are stable proteins, and m909 should have a relatively long half life in circulation, providing an option when prolonged treatment targeting FRβ is desired.

m909 mediates ADCC in FRβ-positive cells, suggesting that it could be used to eliminate activated macrophages or AML cells as a monotherapy without the need to couple it to drugs. Our experiments showed that there is a significant amount of m909-bound FRβ on CHO-FRβ cell surfaces after incubation at 37°C for 1 hour. Many surface receptors undergo downmodulation upon antibody binding. The fact that the intensity of m909-bound receptor remains strong after 37°C incubation implies that the receptor internalization is slow or represents a small fraction of the receptor or that recycling and rebinding overwhelm the internalization. In any case, the presence of m909-decorated receptor at high levels allows time for NK cells and macrophages to kill these disease cells.

Because m909 and folate do not interfere with each other's binding on FRβ (Figure 4c, d), m909 and folate-drug conjugates may be used in combination to increase efficacy. This feature of m909 is also important for m909 monotherapy to work in a clinical setting because, in the serum of healthy people, there is a significant level of free folate (18.2 μg/L or 42 nM on average) [29]. Also, binding of m909, unlike folate-drug conjugates, will not be affected by the folate levels in the system.

m909 was selected from a naïve human antibody library and its sequence does not deviate significantly from the germline sequences. The V gene of m909 heavy chain has 98.61% identity to its closest germline gene IGHV1-3*01, and the V gene of the light chain has 96.77% identity to IGLV3-19*01. The close homology of m909 to germline genes supports a possibility that it will be well tolerated by the immune system. Finally, because no high-affinity FRβ-specific antibody is commercially available, m909 can be used as a research reagent to study the function of FRβ.

## Conclusions

m909 has approximately 57 nM affinity in Fab form and femtomolar avidity in IgG1 form. Unlike folate-drug conjugates, m909 selectively binds to FRβ, does not recognize FRα, and has at least one effector function. m909 alone has the potential to eliminate FRβ-positive cells. Because m909 does not compete with folate for receptor binding, it can be used with folate-drug conjugates in a combination therapy. m909 can also be a valuable research reagent.

## Abbreviations

ADCC: antibody-dependent cell-mediated cytotoxicity; AML: acute myelogenous leukemia; EC_50_: half maximal effective concentration; ELISA: enzyme-linked immunosorbent assay; Fab: antigen-binding fragment; FITC: fluorescein isothiocyanate; FR: folate receptor; FRβ: folate receptor beta; GPI: glycosylphosphatidylinositol; hFRβ: human folate receptor beta; K_D_: equilibrium dissociation constant; mAb: monoclonal antibody; NK: natural killer; PBMC: peripheral blood mononuclear cell; PBS: phosphate-buffered saline; PBST: 0.05% Tween 20 in phosphate-buffered saline; PCFT: proton-coupled folate transporter; RFC: reduced folate carrier; rFRβ: recombinant folate receptor beta; scFv: single-chain variable fragment.

## Competing interests

PSL has received fees and stocks from Endocyte Inc. (West Lafayette, IN, USA), a company that he founded in 1995 to develop treatments for cancer. Because Endocyte plans to develop drugs (but not antibodies) for treatment of autoimmune and inflammatory diseases, this relationship could constitute a conflict of interest. The National Institutes of Health and Purdue University have applied for a patent claiming m909; however, the authors receive no benefits from the patent application. The authors declare that they have no other competing interests.

## Authors' contributions

YF helped to initiate the study, carry out experiments in different areas, analyze data, and write the manuscript. JS helped to carry out experiments in different areas, analyze data, and write the manuscript. EDS helped to carry out experiments in different areas. ML provided patient samples. ZZ provided the library. PSL and DSD helped to initiate the study, analyze data, and write the manuscript. All authors read and approved the final manuscript.
